# Editorial: Unraveling inflammaging: a pathway to prevent age-related disease in animals, volume II

**DOI:** 10.3389/fvets.2026.1835614

**Published:** 2026-05-08

**Authors:** Toshiro Arai

**Affiliations:** School of Veterinary Medicine, Nippon Veterinary and Life Science University, Musashino, Japan

**Keywords:** age-related disease, inflammaging, preventive medicine, senescence, serum amyloid A

The occurrence of various age-related diseases increases in aged animals. Aging is characterized by chronic systemic inflammation accompanied by cellular senescence, immunosenescence, and organ dysfunction. A chronic low-grade pro-inflammatory state known as “inflammaging” accelerates age-related diseases such as obesity, diabetes, vascular diseases, and certain types of cancer. Senescent cells drive age-related tissue dysfunction partially by inducing a chronic senescence-associated secretory phenotype (SASP) associated with various diseases. Cellular senescence is a state of permanent cell proliferation arrest induced by persistent DNA damage and other stress-induced signals. Many senescent cells secrete a wide spectrum of bioactive factors, including inflammatory cytokines, chemokines, growth factors, matrix metalloproteases, lipids, nucleotides, extracellular vesicles, and soluble factors, termed SASP. The SASP has been proposed as the underlying cause of inflammaging and comprises various soluble factors, including proinflammatory mediators and matrix-degrading molecules characterized by the release of pro-inflammatory cytokines. Inflammaging is characterized by increased blood levels of several inflammatory biomarkers, including CRP, IL-6, IL-8, and TNF-α.

Age-related changes in body composition and physical fitness (physical frailty, physical inactivation, ectopic lipid disposition, visceral adiposity, insulin resistance, and SASP) are among the most apparent and unavoidable effects of aging and cause metabolic dysfunction. In this Research Topic, various metabolic dysfunctions and aberrations of hormone secretion are reported in aged dogs, cats, and horses. Age-related diseases have many causes, and they have no specific nutritional, environmental, or pharmacological interventions. As prevention of severe diseases is required, early diagnosis of age-related diseases is very important. The development of new diagnostic biomarkers for inflammaging could lead to early diagnosis and prevention of age-related disease. In aged animals, the incidence of age-related diseases is inevitable. However, the extension of healthy life years and the ability to stay healthy until old age are standard medical requirements. Therefore, the early diagnosis of inflammaging could be a significant step toward preventing various age-related diseases in animals. Serum amyloid A (SAA) is used as an acute phase protein for acute inflammation in cats, and SAA is reported to be potentially useful as a chronic inflammation marker (inflammaging marker) in obese cats.

This Research Topic also considers the critical role diet plays in either exacerbating or mitigating the inflammatory state associated with aging. Nutrition is a key factor that can influence the degree of inflammaging through various mechanisms, including the modulation of gut microbiota, antioxidant intake, and fatty acid profiles. Proper nutritional strategies could potentially attenuate the proinflammatory environment by enhancing the body's ability to counteract oxidative stress and improving metabolic health. Investigating the impact of specific nutrients, dietary patterns, and feeding regimes on the onset and progression of inflammaging can provide valuable insights, leading to the development of dietary interventions aimed at promoting healthier aging in animals. Thus, this Research Topic intends to bridge the gap between animal nutrition and inflammaging, contributing to the broader understanding of how dietary factors affect age-related inflammatory processes and overall healthy life year in animals.

Various phytochemicals have been developed as senolytic drugs. Resveratrol, a natural polyphenol found in plants such as peanuts, grapes, and strawberries, modulates the expression of pro- and anti-apoptotic factors, neutralizes free radical species, affects mitochondrial function, chelates redox-active transition-metal ions, and prevents protein aggregation. Resveratrol inhibits the SASP through the SIRT1/NF-κB signaling pathway and delayed aging. Early diagnosis of the SASP by detecting various pro-inflammatory cytokines and inflammatory markers is possible. In cats with age-related obesity, SAA can be a good diagnostic marker at an early stage of inflammaging. Aging is inevitable in animals, however, delaying the onset of age-related diseases through adequate interventions in the early stage of the SASP is possible. Adequate nutrition, moderate exercise, and a good mental state can effectively prevent age-related diseases, including obesity, in cats. Alleviation of inflammaging will help prevent age-related diseases. Moreover, stem-cell therapy is effective at lessening the severity of some kinds of age-related diseases in dogs and cats.

Age-related diseases become more common as animals live longer, and alleviation of inflammaging can prevent severe age-related diseases. More research on inflammaging may reduce the incidence of age-related diseases in animals.

